# A Scoping Review Mapping Research on Green Space and Associated Mental Health Benefits

**DOI:** 10.3390/ijerph16122081

**Published:** 2019-06-12

**Authors:** Charlotte Wendelboe-Nelson, Sarah Kelly, Marion Kennedy, John W. Cherrie

**Affiliations:** 1 Institute of Biological Chemistry, Biophysics and Bioengineering, Heriot Watt University, Edinburgh EH14 4AS, UK; j.cherrie@hw.ac.uk; 2 Information Services, Heriot Watt University, Edinburgh EH14 4AS, UK; S.Kelly@hw.ac.uk (S.K.); m.l.kennedy@hw.ac.uk (M.K.); 3 Centre for Human Exposure Science, Institute of Occupational Medicine, Research Avenue North, Edinburgh EH14 4AP, UK

**Keywords:** green space, mental health and wellbeing, exposome

## Abstract

Background: There is a growing interest in research investigating the association between green space (GS) and mental health and wellbeing (HWB), in order to understand the underlying mechanisms. Accordingly, there is a need to map the literature and create an overview of the research. Methods: A scoping review approach was used to map literature on GS, including context and co-exposures (the GS exposome), and their associations with mental HWB. The review considers mental HWB definitions and measurements and how GS is characterized. Furthermore, the review aims to identify knowledge gaps and make recommendations for future research. Results: We identified a great diversity in study designs, definitions, outcome measures, consideration of the totality of the GS exposome, and reporting of results. Around 70% of the 263 reviewed studies reported a positive association between some aspect of GS and HWB. However, there is a limited amount of research using randomized controlled crossover trails (RCTs) and mixed methods and an abundance of qualitative subjective research. Conclusions: The discords between study designs, definitions, and the reporting of results makes it difficult to aggregate the evidence and identify any potential causal mechanisms. We propose key points to consider when defining and quantifying GS and make recommendations for reporting on research investigating GS and mental HWB. This review highlights a need for large well-designed RCTs that reliably measure the GS exposome in relation to mental HWB.

## 1. Introduction

Several reviews have highlighted the positive association between green space (GS) and mental health and wellbeing (HWB). These reviews have generally focused on GS in a narrow sense, such as forest therapy [1,2], community GS [3,4], or urban GS [5,6,7], and a number of reviews have looked at GS in relation to urbanicity and urban planning [8,9]. Other reviews have focused on specific GS activities, such as community gardening [10], horticultural therapy [11,12], therapeutic gardening for the elderly, [13], spending time in a forest [2,14], and GS in the living environment [15]. Reviews have also explored the connections between biodiversity, ecosystem services, and human health and wellbeing [16,17,18]. The reviews generally identify positive associations between the narrowly defined GS investigated and measures of mental HWB.

Design of and access to GS is particularly relevant in cities where GS, among other social and environmental factors, is under pressure due to urbanization [19]. It is estimated that by 2050, more than two-thirds of the world’s population will live in urban areas. This has led to a large number of research studies with a focus on mental HWB and access to urban GS. Urbanization is associated with increased levels of mental illness, including anxiety and depression [20,21,22]. Access to urban GS has been positively associated with mental HWB [23,24], but the underlying reasons for this are still not well-understood.

GS has also been shown to be associated with mental HWB in rural areas [25,26,27,28]. When Gilbert, Colley and Roberts [29] investigated subjective wellbeing in rural areas of Scotland, they found that residents living in remote rural areas reported higher levels of life satisfaction compared with non-rural areas. Other studies investigating associations between mental HWB and GS in rural areas have found a significant relationship with rurality [30,31].

Botanical gardens have been proposed as alternative ways to stay in touch with nature [32,33,34]. A number of studies have shown a positive relation between garden/horticultural therapy and a number of psychological issues, e.g., stress management [35,36,37,38], treatment of depression [39,40,41], rehabilitation of prison inmates [42], and wellbeing among elders [43,44,45].

There is increasing interest in understanding the factors that may make GS beneficial for HWB [46]. However, most reviews do not consider contextual factors, such as culture and accessibility, or co-exposures, such as sound and light. The developing concept of the exposome [47] encompasses the totality of exposures we face as humans, from conception onwards, and the combined effect of these exposures on HWB. An exposome approach to investigating GS could help us understand exactly what is beneficial for mental HWB.

We have carried out a scoping study to map the available literature on different types of GS, including the context and co-exposures, and their associations with mental HWB, considering how mental HWB is defined and measured and how GS is characterized. Furthermore, the review aims to identify any current knowledge gaps and make recommendations for future research on the subject.

## 2. Materials and Methods

A five-step scoping review methodology was used to collect, evaluate, and present the analysed literature [48]:

- Identifying the research question(s);
- Identifying relevant studies;
- Study selection;
- Charting the data;
- Collating, summarizing, and reporting the results.

The following research questions (RQ) were used to underpin the search strategy:

1. How do different types of GS (recreational, residential, urban, rural) affect HWB and how much green space is needed for health improvement?
2. How can we best define, measure, and quantify GS and mental HWB?
3. Do different co-exposures or contextual factors affect the mental HWB outcome?
4. Do different age groups and population subgroups benefit differently from exposure to GS?

Theoretical, empirical, and experimental studies were included, with a focus on links between GS of any description and mental HWB of any definition. To our knowledge, no review has attempted to map the totality of literature on GS and the associated effects on mental HWB. In this scoping review, we adopt a wide definition of GS and GS activities, including small urban pockets of GS, remote rural areas, horticultural therapy, allotment gardening, and virtual green space. This was done to try to shed light on the effect of contextual factors and co-exposures potentially influencing the effects of GS on mental HWB.

Studies with a main emphasis on biological diversity or physical activity, not including a detailed investigation of associated mental HWB outcomes, were excluded. Studies focusing on children under the age of 18 were excluded, as the mechanisms and contextual factors related to mental HWB may be different in children than in adults. In situations where the age range of participants included people under the age of 18, a decision to include or exclude the paper was based on each individual study, considering the contribution the study findings and conclusions would make to this review. Studies with an emphasis on GS in war or disaster zones were excluded, as these are extreme circumstances and not applicable to the general population. Studies with a focus on urban design, not investigating any associated mental HWB outcomes, were also excluded. Only peer-reviewed literature was included, and grey literature and all conference proceedings, abstracts, or opinion pieces were excluded. Keywords for two main concepts were generated and used for the literature search (Table 1).

Relevant studies and literature reviews from peer-reviewed journals were identified using Web of Science, MEDLINE, PsycINFO, PubMed, Scopus, GreenFILE, and SPORTDiscus. Additionally, research evidence was sought from topic-related networks and relevant organizations, and reference lists of earlier key studies were used to detect relevant publications not identified in the original main search [48].

**Figure 1:**
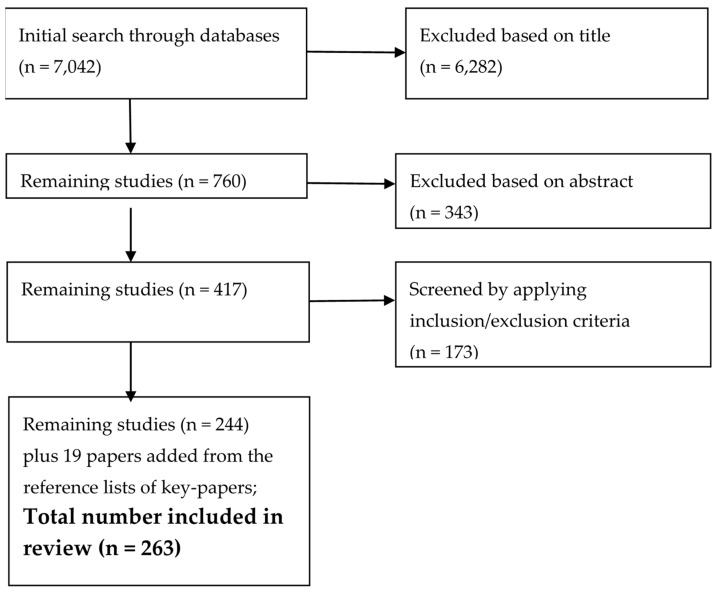
Article screening and selection process.

All papers were pooled and duplicates removed, resulting in a total of 7042 papers. The literature was initially screened by two members of the research team (CWN, JC), using a comparative and consensus orientated method (Figure 1). After exclusion based on the title and abstract, there were 417 papers for review. When applying the inclusion/exclusion criteria, another 173 papers were excluded. An additional 19 papers were included from the reference lists from key-papers, taking the total papers for review to 263.

The included literature was charted following the technique described by Arksey and O’Malley [47], to synthesize and interpret the studies by sorting them according to key issues and themes. Each study was analysed according to the type of GS investigated, health outcomes and measures, experimental design, and methods used. The quality of the included studies was not systematically assessed, so this review does not determine the robustness of findings from the included literature. The reviewed literature was then collated, summarized, and reported in four thematic groups (Table 2).

The literature was further divided into ‘type of study’ (cross-sectional or longitudinal, controlled trial, randomized or non-randomized, with or without crossover); ‘methods’ (what methods have been used to measure mental HWB and GS. Quantitative or qualitative data collection methods); ‘health outcome’ (the type of mental HWB assessed); and whether the study has reached a positive or negative conclusion (were initial hypothesis proven right or wrong). Comprehensive lists were generated, comprising all the different mental HWB outcomes investigated and all the different tools used to assess the health outcomes. This was done to get an overview of the totality and complexity of studies, and to identify the most commonly used methods for assessing mental HWB.

## 3. Results

### 3.1. Numerical Analysis

This analysis is used to highlight the dominant areas of research with respect to the study design, type of participants, methods used, main conclusions, and country where the study has been conducted. The papers were divided into groups based on the study design (Table 3). The majority of studies were cross-sectional (86.3%), with only 13 studies being longitudinal (4.9%). There were nine studies with a Randomized Controlled Trial (RCT) study design with a crossover element and 21 studies using an RCT study design without a crossover element.

The majority of studies used only qualitative methods 212 (80.6%), with only 29 studies using a combination of qualitative and quantitative data collection methods (11%). Twenty-two of the publications were reviews (8.4%).

Different countries will face different co-exposures and contextual factors, which may potentially affect the HWB outcomes in different ways (RQ 3). To understand the representation from around the world, the literature was charted according to the continent where the study took place (Table 4). The majority of studies were conducted in Europe (46.8%), followed by North America (24.3%), Asia (11%), and Australia (6.8%). Most of the studies conducted in Europe were from western and northern parts; the UK (38%), followed by Sweden (15.4%) and the Netherlands (6.5%). Based on Table 4, it is evident that a majority of studies have been carried out in the developed part of the world. The identified benefits of GS in developed countries may not be applicable to less developed countries. The same is the case between temperate and tropical areas, with most studies being carried out in the former.

Different population subgroups might benefit differently from exposure to GS (RQ 4). To investigate what population subgroups have typically been used to assess the effects of GS on mental HWB, the literature was sorted according to participant type (Table 5). For ease of overview, the different participant types have been grouped together where reasonable overlap and similarity was identified. The most common type of study participant was the general public (30% of all included studies), followed by university and college students (14.1%) and individuals with mental health issues and disorders (12.2%). There is a long list of studies that have used more specific participant types, i.e., park users, allotment gardeners, adults with burnout syndrome, depression, mental health issues, female prisoners, woodland workers, people building their own houses etc. Therefore, despite the majority of papers focusing on the general public, there is a great variety of specific population subgroups being investigated in relation to the health benefits of various types of GS exposure (Table 5).

Studies were charted as ‘positive’ if the hypotheses were confirmed, ‘negative’ if the main hypotheses were not confirmed, and ‘mixed’ if the hypotheses were only partly confirmed. Note that a study charted as ‘negative’ does not necessarily mean the study found a negative effect of GS exposure on mental HWB. Only 4.6% of studies were charted as negative (see e.g., [49,50]), 25.7% of studies were charted as ‘mixed’, and 70.1% of studies were charted as ‘positive’. It should be noted that a proportion of the studies report a positive finding in the abstract, but when investigating the results in more detail, we found that any mixed or negative findings were played down in the summary. The percentages presented here are based on the abstracts.

### 3.2. Thematic Analysis

The literature has been organized according to thematic groups to address research question 1. There were 22 literature reviews identified, which are not included in this thematic analysis. An in-depth evaluation of these is beyond the scope of this review.

Group 1 encompasses studies focusing on horticulture, gardens, and allotments (43 studies). Included in this group is garden or horticultural therapy, and access and use of private and public gardens. The literature in this group was further divided into seven categories: private gardens (4.6% of Group 1 studies), complex interventions (14%), allotments (18.6%), horticultural therapy (30.2%), occupation (4.7%), public gardens (18.6%), and community gardening (9.3%). Out of these 43 studies, only seven had a quantitative element [45,51,52,53,54,55,56]. A range of quantitative measurements were used, such as salivary alpha amylase (sAA) levels, an electrocardiogram (ECG), a surface electromyogram (sEMG), a respiration rate, body composition, physical functional ability, hand function ability, BMI, cortisol, sick leave status, and healthcare consumption.

Group 2 encompasses studies focusing on urban GS or mixed GS (140 studies). Included in this group was any GS located in an urban setting, and studies that used a mixture of GS types where it was not possible to assign the study to one of the other groups and where there was a main focus on urbanicity. This group is large and very diverse and for many of the studies, it was difficult to categorize and determine exactly what type of GS was being investigated, due to the lack of details used to describe the space. It was therefore not practical to further divide this group into subgroups in a meaningful way. Out of the 140 studies focusing on urban green space, 130 were cross-sectional and 10 were longitudinal, 125 studies used qualitative methods, and 15 used quantitative or mixed methods.

Group 3 encompasses wild, natural, and rural GS (34). This group includes GS types such as care farms; adventure therapy; rural neighborhoods; and wild nature like mountains, national parks, beaches, and large forests. Due to the diversity of the investigated GS, this group was further divided into eight subgroups: care farms (5.9%), forest GS (29.4%), natural green exercise (2.9%), nature connectedness and restorativeness (8.8%), nature interventions (17.6%), occupational (5.9%), rural communities (11.8%), and wild camping and adventures (17.6%). Out of these 34 studies, 32 were cross-sectional, one was longitudinal, and one study was a secondary narrative analysis. Qualitative data collection methods were used in 27 of the studies, with only seven of the studies using quantitative or mixed methods [1,57,58,59,60,61,62]. A range of objective quantitative data collection methods were used, such as cortisol measurements, cytokine serum levels, blood pressure, and heart rate variability.

The last group, Group 4 (24), includes virtual and indoor GS, e.g., photos, images, videos, and any type of GS enclosed under a roof. This group can be further subdivided into four groups: assessment by questionnaire only and no exposure to GS (50%), indoor GS exposure (8.3%), video of GS (4.2%), and images or photos of GS (37.5%). None of the studies in this group included a quantitative element; 22 studies relied on questionnaire data and two studies have used interviews. Out of the 24 studies, 23 were cross-sectional, with only one study being described as longitudinal [63]. Erikson, Westerberg and Jonsson [63] investigated a therapeutic gardening program taking place in a greenhouse; however, the longitudinal aspect of the study only stretched over three months.

The type of health outcome investigated varied greatly between the included studies. The total number of primary mental HWB outcomes observed and the number of times each outcome has been investigated were summarized (Table 6). Mental health (37), wellbeing (35), and stress (34) were the most used mental HWB outcomes. These were followed by restorativeness (22), depression (19), quality of life (13), psychological wellbeing (12), general health (11), and mental wellbeing (8). It is likely that some of these outcomes are intended to cover the same aspect of mental HWB. However, as a clear definition of the health outcome is rarely presented, it is not possible to confidently and accurately combine these outcomes and group them into fewer groups.

The number of tools used to measure mental HWB and the number of times each tool has been used are summarized in Table 7. Despite the availability of a vast range of validated tools developed to investigate mental HWB, the most common approach was to develop new questionnaires (DOQ; 15.8% of the studies). The most used validated questionnaire was PRS (7.9%), closely followed by PANAS (7.1%), PSS (6.6%), GHQ (6.2%), PS (5.8%), WEMWBS (5.4%), and HS SF-36 (4.1%), and the abbreviations are listed in Table 7.

Table 8 gives an overview of the different tools and what health endpoints the tools have been used to investigate. Some studies have not used tools such as questionnaires, surveys, scales, or inventories, but have instead used interviews, focus groups, observations, or similar methodologies. These studies are not included in Table 8. To measure the ten most used health endpoints (Table 6), the following tools have been most frequently used (Table 9): The GHQ was used in 15.4% of studies investigating mental health; 11.9% of studies developed their own questionnaires (DOQ) when investigating wellbeing; the PSS was used in 13.6% of studies investigating stress; the PRS was used in 32.4% of studies investigating restorativeness; 13.9% of studies used BDI when investigating depression; 16.7% of the studies developed their own questionnaires (DOQ) when investigating quality of life; 8.7% of studies DOQ when investigation psychological wellbeing; the GHQ was used in 17.6% of studies investigating general health; the WEMWBS was used in 21.4% of studies investigating mental wellbeing; and 75% of studies DOQ when investigating life satisfaction. Some studies have used several tools to measure one health endpoint.

### 3.3. Analysis of Study Design

When testing a research hypothesis, an RCT is the most scientifically rigorous method available [359]. In an RCT, the participants are randomly assigned to one of at least two groups; a design that specifically reduces selection bias and is often considered the gold standard for research designs, when considering the efficacy of different treatments compared to a control.

There were 30 RCTs identified; 11.4% of the total number of papers selected for review. Nine (30%) of these studies included a crossover element: eight had a 2-arm design and one study had a 4-arm design. Out of the 21 RCT without a crossover element, ten had a 2-arm design, seven had a 3-arm design, and four had a 4-arm design. Eight studies used a non-randomized Controlled Trial (CT), with a 2-arm design. Two of these studies used a crossover element, and six studies had no crossover element.

It is not always convenient or possible to introduce randomization. In their study, Sung and colleagues [61] evaluated the health effects of a forest therapy program using what they call a ‘convenient assignment’ and not true randomization, which considers the subjects’ preference and suitability to the intervention or the control group. Bang et al. [1] investigated the effects of a forest-walking program on physical and psychological health using a quasi-experimental design. The participants were assigned to the experimental or control group based on the participants’ preference, to boost motivation. Dewi et al. [52] also used a quasi-experimental design, investigating already existing community garden activities. Beute and de Kort [201] investigated if lower mental health makes an individual more or less responsive to the positive health effects of GS. Accordingly, the participants were not randomized, but split into groups based on their obtained score from the BDI-II, which was an appropriate design to answer their particular research question. Non-randomized study designs like these [1,52,61] may say something about the effect of an intervention or activity on people with a predisposition for the environment chosen, which might not represent a result that is transferable to the general population.

There may be other practicalities preventing the use of randomization. Park et al. [54] used a quasi-experimental design with a non-equivalent control group; the groups being two senior community centres, with one participating in a gardening intervention, while the other one did not. Wood and colleagues [321] investigated the health and wellbeing benefits of allotment gardening, using a case-control study to compare allotment gardeners with non-gardeners. In many real-life situations, such methods [54,321] will be the only possible way to evaluate an intervention and randomization is not an option. However, if the process, context, and delivery of the intervention are considered, this type of evaluation may produce meaningful results.

Another aspect that can increase the rigidity of a study design is the incorporation of a crossover element [360]. In a study with a crossover design, all participants receive both the intervention treatment and the control treatment. The different treatments are given at different times and with a sufficient washout period in between to insure there is no carryover effect from one treatment into the next. The order of the treatments is randomized. When using a crossover design, the between-subject variability is significantly reduced as each participant serves as their own control. This results in a reduction of the variation in factors not related to the treatment, which in turn allows for the detection of smaller effect sizes using a reduced sample size [360]. However, crossover designs need careful design to minimize potential bias.

Barnicle and Midden [289] investigated the effects of a horticultural activity program on psychological wellbeing among older people in two care homes. As randomization would not be practical at the individual level, the randomization took place at the site level. It would, however, have strengthened the study design if a crossover had been introduced and participants from both care homes had been exposed to the intervention and the control treatment. The authors give no explanation as to why they chose not to include a crossover. However, this often comes down to time, funding, and the likelihood of being able to secure participation and retention for an extended period of data collection. A number of studies fall into this category; an RCT study design that would have benefitted significantly from a crossover element introduced to the design (see, for example, [202,306,319,320,324,335]).

Seven studies identified for this review used a 3-arm design; typically, two intervention treatments and one control treatment [304], or three different types of intervention treatment [282,336]. None of these 3-arm RCTs have incorporated a crossover element. This is not unexpected, as adding more arms to a study design will increase the complexity of the study and put strain on resources, such as time, money, and by no means least the participants.

Five studies used a 4-arm RCT. Sonntag-Ostrom and colleagues [310] investigated the restorative effect of visits to one urban area and three different forest environments, with each participant visiting all four outdoor environments. The authors highlight the difficulties in carrying out a study with such a complex design, e.g., a long data collection period and difficulties in recruiting participants. These difficulties resulted in a 3-year project and only 20 participants [310].

Based on the studies included in this review, the strongest design appears to be RCTs with a crossover element, a finding which is also supported in other literature [359,360]. In addition, the results from this review highlight that unless answers to very specific research questions are sought, increasing the complexity of the study design does not necessarily improve the quality of the data collected as constraints and limitations increase with increasing complexity.

There were eight studies using a 2-arm RCT with a crossover element (Table 10). Three of the studies focused on urban GS, four on natural GS, and one study on virtual/indoor GS. Six of the studies were qualitative, and only two studies used qualitative as well as quantitative methods. Seven of the studies predominantly used questionnaires as the main tool to assess the changes in the investigated health outcome.

Berman et al. [209] used a 2-arm RCT with a crossover to show that participants exhibited a significantly increased memory span after a walk in the park compared to an urban walk. The PANAS (positive affect) revealed a significant effect of location (nature vs. urban) but not time (pre-walk vs. post-walk); for a negative effect, there was no significant effect of location and the negative effect did not decrease more for the park walk than for the urban walk. The authors were therefore not able to show conclusively that GS positively affects the mood of individuals with depression. Gatersleben and Andrews [50] found that exposure to GS with high levels of prospect (clear field of vision) and low levels of refuge (places to hide) generated a restorative effect. However, the authors also found that exposure to GS with low levels of prospect and high levels of refuge did not create a restorative effect. Such a scenario was proposed to increase stress levels and reduce attention. Im et al. [57] found that the levels of somatic and depressive symptoms, and of stress responses, were significantly reduced after exposure to a forest environment, when compared to exposure to an urban environment. The authors also found a significant reduction of immunological inflammation and an increase in the antioxidant effect after the forest exposure. However, due to the design of the study (no before-and-after measurements allowing for comparison), it is not clear if the positive changes are related to a reduction in air pollution (or other harmful urban exposures), rather than the presence of the forest environment. Lee et al. [59] found that the salivary cortisol concentration, diastolic blood pressure, and pulse rate were all significantly lower in participants after exposure to a forest environment. Self-reported subjective measures revealed that participants felt more comfortable, soothed, and refreshed when viewing a forest landscape, when compared to an urban environment. Morita and colleagues [326] investigated the psychological effects of exposure to a forest environment, when compared to exposure to a control environment. Co-exposures and contextual factors were considered, such as conditions during the forest visit and on the control day (weather, duration of visit, previous visits, accompanying people, activities undertaken, walking course and distance walked, degree of exercise, subjective feelings, objective activities undertaken). The authors found that exposure to a forest environment significantly decreased feelings of hostility and depression, and increased the feeling of liveliness, when compared to exposure to a control environment. It was also seen that the positive effect of exposure to a forest environment was greater the higher the stress level of the subject. Despite a high number of participants and a generally stringent study design, the study only used qualitative data and would have benefited from the inclusion of quantitative data. South et al. [361] found that when subjects were in view of a green vacant lot, their heart rate decreased significantly, when compared to being in view of a non-greened vacant lot or not in view of any vacant lot. The authors conclude that remediating neighborhood blight can reduce stress and improve health. Takayama et al. [233] investigated the emotional, restorative, and vitalizing effects related to forest and urban exposures and concluded that exposure to a forest environment improved mood and positive affect, and induced a feeling of subjective restoration and subjective vitality. Tenngart Ivarsson and Hagerhall [318] investigated the perceived restorativeness of gardens. Two gardens with differing levels of build and natural elements were photographed, and a set of 12 photos were selected to represent each garden. The PRS was used to examine the perceived restorativeness of the two gardens. The study also aimed to evaluate the ability of the PRS to distinguish between two different gardens with a mix of build and natural elements, rather than to distinguish a contrast between built and natural scene types. The authors found that both gardens were perceived as restorative, and the PRS can be used to discriminate between two gardens from the same scene type. Hence, one garden can be perceived as more restorative than another although they both have the same type of scene. This highlights the importance of considering the contribution of contextual factors and co-exposures to the overall health effect caused by a GS environment.

Out of the eight studies included here, with a 2-arm RCT crossover design, seven had a positive outcome. Only two of the studies included quantitative measures [57,59], with both studies having a low participant number. All studies heavily rely on qualitative subjective data (Table 10), on which it is difficult to draw comparative conclusive interpretations. However, it is evident that in the included RCTs, there is clear agreement of a positive association between GS exposure and mental HWB. Despite the lack of high-quality studies and methodological rigor between studies, the accumulated strength of these findings highlights the importance of the positive associations between GS and mental HWB.

## 4. Discussion

The effects of GS on mental HWB is relevant to city planning and public health policy, which is becoming increasingly important as the world’s urban population grows. The published research generally shows positive associations between GS and mental HWB. However, this review has identified great diversity in study designs, GS definitions, outcome measures, inclusion of co-exposures and contextual factors, and reporting of results. This makes it difficult to aggregate the evidence to identify the underlying mechanisms for this positive association or to provide advice to help construct GS that is beneficial for mental HWB.

Based on the diversity of research available on the subject, it is not possible to unequivocally answer all of the four research questions we initially posited. However, based on the weight of evidence of the research reviewed, it is possible to conclude the following with reasonable certainty:

- Different types of GS in many contexts and different environments have a positive effect on mental HWB (RQ 1 & 3);
- For a variety of different groups of people (RQ 4), GS does have a positive effect on mental HWB;
- Different types of GS affect the HWB of individuals in different ways (RQ 1 & 4).

However, based on the analysed literature it is clear that there is no universally agreed definition for GS or mental HWB and in many studies, a definition and/or detailed description of the two has been omitted. Only a few studies have attempted to quantify the GS investigated and/or the amount of GS needed for health improvement (RQ 1 & 2).

RQ 1: How do different types of GS (recreational, residential, urban, rural) affect HWB and how much green space is needed for health improvement?

There are suggestions that different types of GS may affect mental HWB in different ways and that different age groups and population subgroups benefit differently from exposure to GS. There is also limited evidence that some threshold amount of GS is needed to generate positive health outcomes. However, there is insufficient coherence in the evidence to generalize the results.

RQ 2: How can we best define, measure, and quantify GS?

Often, the description of the GS is limited to simple text descriptors, e.g., allotment garden, urban park, or private garden. There are some good examples of studies that have attempted to quantify the GS investigated and assess the GS quality. For example, Tilley et al. [362] included graphic Ordnance Survey maps clearly depicting the urban environments investigated, giving a clear overview of the settings and contexts. A written overview and typology was included, of quartiles of urban green and urban busy areas, derived from a Geographic Information System (GIS). The authors also used photographs giving visual evidence of the different environments, which would make it easy to replicate the study in other cities and countries. Our findings highlight the necessity to investigate further how best to define, measure, and quantify GS. With a systematic review, it would be possible to explore in more detail what types of measurements are used most efficiently to quantify GS, the accuracy of the different methods, and the reproducibility.

RQ 2: How can we best define, measure, and quantify mental HWB? The World Health Organisation (1948) has defined health as “A state of complete physical, mental and social wellbeing and not merely the absence of disease and infirmity”. However, wellbeing is difficult to define. Fleuret and Atkinson [363] reviewed the various ways in which wellbeing has been used in research and policy contexts. They note that the term ‘wellbeing’ mainly originates from Anglophone countries and in many languages, it is difficult to find and appropriate comparable terms. Often, a number of different terms are used interchangeably to describe wellbeing, such as quality of life, happiness, welfare, pleasure, wealth, and subjective and objective wellbeing [363]. These terms are rarely specified, and it is therefore impossible to know if they are synonymous. Additionally, different stakeholders in different countries adhere to the wellbeing concept in various ways and it is a matter of practice amongst stakeholders that determines how a term is defined. As far as possible, it would be an advantage to harmonize definitions of HWB and to at least explicitly describe the definition used in a research study. The definition proposed by The UK Faculty of Public Health is perhaps a good starting point:

- ‘Realise our abilities, live a life with purpose and meaning, and make a positive contribution to our communities;
- Form positive relationships with others, and feel connected and supported;
- Experience peace of mind, contentment, happiness and joy;
- Cope with life’s ups and downs and be confident and resilient;
- Take responsibility for oneself and for others as appropriate.’

(Faculty of Public Health, 2010: https://www.fph.org.uk/policy-campaigns/special-interest-groups/special-interest-groups-list/public-mental-health-special-interest-group/better-mental-health-for-all/concepts-of-mental-and-social-wellbeing/).

This holistic definition of wellbeing incorporates a more social aspect, highlighting a change in focus from looking more at physical health to looking at the realization of the individuals’ potential [364]. It is more inclusive and relevant to more diverse population subgroups, such as people with learning disabilities, who in many cases experience chronic conditions on a daily basis [365]. Furthermore, we propose that the quality of the environment, i.e., built or natural, is also taken into consideration when assessing wellbeing in such a holistic way, in line with the GS exposome.

RQ 3: Do different co-exposures or contextual factors affect the mental HWB outcome?

Very few studies included in this review have taken contextual factors and co-exposures into account; they were generally poorly described and so it is difficult to replicate studies. The importance of this is highlighted in a study by McMahan and Estes [46], who aimed to synthesize research on the effect of exposure to natural environments on positive and negative affect, using a meta-analysis technique. The authors only included studies with an RCT design including a comparison group and a self-report assessment of the current emotional state; 32 papers were identified. Study and design-related characteristics, such as the year of publication, location of study, mean age of sample, percent female, and instrument used to measure affective wellbeing, were examined to reveal if they had a moderating effect on the investigated outcome. The type of exposure was also addressed (i.e., real or laboratory simulations of nature), as was the type of natural environment (i.e., manicured or wild nature). The review concluded that exposure to natural environments was associated with a moderate increase in positive affect and a small decrease in negative affect. The authors found that study location, type of assessment used to measure emotion, and type of exposure moderated the effect of nature on positive affect. This indicates that co-exposures and contextual factors may play a role in mediating positive as well as negative health effects associated with GS exposure. The attempt in this review, to look at context and co-exposures, has highlighted a gap in the available literature; our knowledge on contextual factors and co-exposures in relation to the GS experience (GS exposome) is insufficient and research is needed to investigate the totality and combination of exposures related to GS that affects mental HWB.

RQ 4: Do different age groups and population subgroups benefit differently from exposure to GS?

Participant type varied greatly between studies and in many cases, the subjects were very specifically specified, e.g., park users, allotment gardeners, or active walkers. These groups may have an affinity for the GS being investigated. This makes it difficult to compare study results and hinders the interpretation of whether a finding can be generalized to other groups within the population. However, based on the weight of evidence, it can be concluded with reasonable certainty that different population subgroups will benefit differently to a variety of GS exposures.

Based on the analysis in this review, we suggest a number of key points that should be assessed and reported when investigating GS exposures:

1. Quantity of greenery or natural elements;
2. Type of vegetation (creating shade or not/natural daylight);
3. Whether the environment is natural or managed;
4. Quantity of built elements;
5. Traffic noise and air pollution levels;
6. General soundscape;
7. Number of people present in the environment;
8. Setting and context.

The majority of studies rely on qualitative data collection methods and there is limited methodological consistency between studies. There is a need for more robust quantitative data collection methods, e.g., using vegetation cover maps from airborne hyperspectral and light detection and ranging (LiDAR) data to derive measures of GS [253], or measurement of stress hormones (cortisol) for the quantification of changes in stress levels after exposure to different urban and natural environments [58,59,61,62,334,335], or in relation to neighborhood GS and long-term exposure [208,329,332,366]. Ng, et al. [367] recently published the findings from an RCT (waitlist-control randomized controlled trial) investigating the effects of horticultural therapy on Asian older adults. Qualitative measures (MOCA, Zung Self-Rating Depression and Anxiety Scales) were used to investigate cognitive functioning, depression, anxiety, psychological wellbeing, and positive relations with others. Quantitative measures were used to measure nine plasma biomarkers ranging from interleukins and chemokines to hormones. Ng, et al. [367] found no significant changes in conventional psychological subjective measures of health and wellbeing after 6 months of horticultural therapy. However, there was a significant reduction in pro-inflammatory cytokines after the intervention; high levels of these cytokines are associated with depression [368]. This highlights the importance of including objective quantitative methods to underpin and clarify any subjective findings.

Recommendations

Overall, we suggest a number of key points that should be included when planning and reporting on findings from research investigating GS and mental HWB:

1. Description of aim and research question(s);
2. Description of the study design;
3. Description of participant type (incl. sex, mean age, min/max age, population subgroup characteristics and other relevant socioeconomic characteristics);
4. Description of recruitment process;
5. Careful description and quantification of the GS investigated (study sites);
6. Clear definition of the mental HWB endpoint(s);
7. Justification of the choice of tools to assess the health endpoint;
8. Measurement of contextual factors and co-exposures.

We advocate that, in future research, the entire GS exposome should be considered when investigating the impact on mental HWB. There is a need for large well-designed randomized controlled crossover trails that reliably measure a range of environmental and personal exposures associated with GS. Future studies should include standardized quantitative data collection methods to describe and define the GS investigated and to quantify the changes in mental HWB. By also including standardized qualitative data collection methods, a meaningful comparison and pooling of data across studies would be possible. This will allow a better understanding of the underlying factors responsible for positive associations between GS and mental HWB.

## Figures and Tables

**Table 1:** Concept 1: Green space. Concept 2: Mental health and wellbeing.

**Concept 1**	
Alternative Terms/Synonyms (combined with OR)	“green space*”, green*, “green environ*”, “green infrastruct*”, outdoor*, “outdoor experience*”, “nature experience*”, “natural space*”, “natural infrastruct*” “green health*”
Broader terms (combined with OR)	“wilderness experience*”, “adventure therapy”, “outdoor therapy”, “nature therapy”, “nature connect*”, “near nature*”, ecotherap*, eco-therap*, “eco therap”, “green therap*”, “green-therap*”, “green exercis*”, “green-exercis*”
Narrower terms (combined with OR)	ecopsychology, eco-psychology, “eco psychology”, “environmental psychology”, park, parks, forest*, horticultur*, “horticulture therap*”, garden*, allotment*, landscap*, highland*, wasteland*
**Concept 2**	
Alternative Terms/Synonyms (combined with OR)	“mental wellbeing”, “mental well-being”, “mental well being”, “mental health”, “emotional wellbeing”, “emotional well-being”, “emotional well being”, “emotional health”, “psychological wellbeing”, “psychological well-being”, “psychological well being”, “psychological health”
Broader terms (combined with OR)	“self-concept”, “self concept”, “self-esteem”, “self esteem”, “self-image”, “self image”, “sense of coherence”, “sense of personal control”, “social wellbeing”, “social well-being”, “social well being”, “psychological issue*”, ruminat*, restorative
Narrower terms (combined with OR)	“well-being”, wellbeing, “well being” “quality of life”, “life satisfaction”, emotion, depress*, anxi*, stress*, fear*, frustrate*, agress*, lonely, loneliness, isolation, happy, happiness, resilien*, optimis*, hope*, empower*

**Table 2:** The literature was divided into thematic groups based on the type of GS investigated (literature reviews are not included).

	Type of Green Space
Group 1	Horticulture, garden, allotment (*n* = 43)
Group 2	Urban and mixed green space (*n* = 140)
Group 3	Wild, natural or rural green space (*n* = 34)
Group 4	Virtual or indoor green space (*n* = 24)

**Table 3:** The included studies were divided into groups based on their study design (some papers are represented in more than one group, i.e., a cross-sectional study with an RCT design).

Type of Study	# of Studies
Cross-sectional	227
Longitudinal	13
Review	22
Historic, secondary narrative analysis	1
Total	263
RCT with crossover	9
RCT, no crossover	21
Non-randomized CT, cross over	2
Non-randomized CT, no crossover	6

**Table 4:** The studies were grouped, based on the continent where the study took place (not including reviews, *n* = 22).

Continent	# of Studies
North America	64
South America	3
Asia	29
South Africa	1
New Zealand	3
Australia	18
Europe	123
-east	5
-west	56
-north	38
-south	6
-central	14
-across regions	4
Total	241

**Table 5:** The literature was charted based on the type of participant included in the study.

Participant Type	# of Studies
General public, parents, twins	79
University students, undergraduates, college students, students, graduate students, university students, healthy and physically inactive, male university students, pupils	37
Psychiatric patients, individuals with clinical depression, mental health patients, stress-related mental health patients, adults with depression, adults with increased psychological stress, adults with mental health issues, individuals with burnout, exhaustion disorder, individual with stress, patients with depression, individuals with burnout, diagnosed with depression/anxiety/stress, females diagnosed with exhaustion disorder, mental disorder clients, individuals with stress injuries, people with mental health problems, people with significant mental ill-health, people diagnosed with chronic mental illness	32
Older adults, over 65’s, elderly women	15
Office workers, science park employees, university office staff, employees, workers	11
Park users, allotment gardeners, recreational walkers, botanical garden visitors, forest users, GS users, greenway trail users, forest users/volunteers	18
Female healthcare workers, health care workers, caregivers, rehabilitation team members, practitioners/decision-makers, public sector employees	6
Athletes, physically active, active runners, experienced runners	6
Dementia sufferers, cancer patients, palliative care patients, individuals with hypertension, chronic stroke patients	7
Deprived communities, vulnerable, homeless women, female prisoners, deprived urban neighborhoods	9
Rural elders, rural population, local residents (predominantly farmers), local residents (farmers and visitors)	4
Adults with disabilities, individuals with disabilities, individuals with learning difficulties, people with disability	4
Postmenopausal women, pregnant women, women	4
Tourists, experienced physically fit backpackers	3
Forest workers, woodland workers	3
African Americans	1
People building houses	1
Alcoholics	1

**Table 6:** The studies were grouped according to the primary mental HWB outcome investigated in the study. Some studies investigate more than one primary outcome.

Primary Mental HWB Outcome	# of Times Used
Mental health	37
Wellbeing	35
Stress	34
Restorativeness	20
Depression	19
Quality of life	13
Psychological wellbeing	12
General health	11
Mental wellbeing	8
Life satisfaction	6
Aggression	4
Affect	3
General wellbeing	3
Anxiety, cognition, emotion, happiness, mood, psychological distress, self-esteem, stress reduction	16 (2 papers for each of the health endpoints)
Chronic stress, clinical depression, emotional wellbeing, general preference for GS, health anxiety, job stress, mental stress, personal development, psychological health, psychological restoration, psychological stress, rumination, severe stress, social integration, stress-related mental illness, stress restoration, stressful life events	17 (1 paper for each of the health endpoints)

**Table 7:** An overview of the tools used to measure mental HWB and the number of times each tool has been used (where the available primary reference for each tool is added in brackets).

Abbreviation	Health Outcome Measure	# of Times Used
DOQ	Developed own questions and questionnaires	38
PRS	Perceived Restoration Scale [64,65]	19
PANAS	Positive and Negative Affect Schedule [66]	17
PSS	Perceived Stress Scale [67]	16
GHQ	General Health Questionnaire [68]	15
PS	Population survey with incorporated health and wellbeing assessments	14
WEMWBS	Warwick-Edinburgh Mental Well-being Scale [69]	13
HS SF-36	Health Survey (SF-36) [70]	10
BDI	Beck Depression Inventory [71,72,73].	9
POMS	Profile of Mood States [74,75,76]	8
CN	Connected to nature [77]	8
STAI	State-Trait Anxiety Inventory [78]	8
CES-D	Centre for Epidemiologic Studies Depression Scale for Research in the general population [79]	7
NCPC	Necker Cube Pattern Control [80]	6
RSE	Rosenberg self-esteem scale [81]	5
K10	Kessler Psychological Distress Scale [82]	5
SWLS	Satisfaction with Life Scale [83]	4
WHOQOL	WHO Quality of Life Questionnaire [84]	4
DASS	Depression Anxiety Stress Scale [85]	4
PHQ	Patient Health Questionnaire [86]	4
SMBQ	Shirom-Melmed Burnout Questionnaire [87]	4
SVS	Subjective Vitality Scale [88]	4
GDS	Geriatric Depression Scale [89]	4
HS SF-12	Health Survey (SF-12) [90,91]	3
EQ-5D	EuroQol-5Dimensions [92]	3
INS	Inclusion of Nature in Self scale [93]	3
ICD	The International Classification of Diseases (WHO)	3
PWB	Psychological Wellbeing Scale [94]	2
QPS	QPSNordic-ADW; Nordic Questionnaire for Monitoring the Age Diverse Workforce [95]	3
BAI	Beck Anxiety Inventory [96]	2
MHI-5	Mental Health Inventory [70,97]	2
MMSE	Mini-Mental state examination (Folstein test) [98]	2
REQ	Recovery Experience Questionnaire [99]	2
RRQ	Rumination-Reflection Questionnaire [100]	2
UWES	Utrecht Work Engagement Scale [101]	2
MAAS	Mindful Attention and Awareness Scale [102]	2
FS	Feeling Scale, affective valence assessed by the FS [103]	2
HAM-17	Hamilton Depression Rating Scale [104]	2
IPA	Interpretative Phenomenological Analysis [105]	2
SHCI	Subjective Health Complaints Inventory [106]	2
LSIA	Life satisfaction inventory A [107]	2
SPNE	Scale of Positive and Negative Experience [108]	2
ZIPERS	Inventory of Personal Reactions, measuring affect [109]	2
PAQ	Place attachment questionnaire [110,111]	2
MSS	Mood Survey Scale [112]	1
GSES	General Self-efficacy Scale [113]	1
CRC-QOL	Instrument developed by [114,115] to measure quality of life	1
TPI	Trier Personality Inventory [116]	1
ABS	Affect Balance Scale [117]	1
AFI	Attentional function index [118]	1
BM	Behaviour mapping [119]	1
BRFSS	Behavioural Risk Factor Surveillance System. (A United States health survey that looks at behavioural risk factors. It is run by Centre for Disease Control and Prevention and conducted by the individual state health departments and is the world’s largest such survey).	1
BF	Big Five [120]	1
BSI	Brief Symptom Inventory (anxiety) [121]	1
BS	Brooding Scale (Rumination) [122]	1
BPAQ	Buss-Perry Aggression Questionnaire [123]	1
CMAI	Cohen-Mansfield agitation inventory [124]	1
CSAI-2	Competitive state anxiety inventory-2 [125]	1
CD-RS	Connor-Davidson resilience scale [126]	1
CS-DD	Cornell scale for depression in Dementia [127]	1
DSI	Daily Stress Inventory [128]	1
DEMQOL	Dementia quality of life instrument [129]	1
SCL-90-R	Symptom Check List [130]	1
ES-SF	Ecology Scale, Short-Form [131]	1
EPDS	Edinburgh postnatal Depression Scale [132]	1
EES	Elevating Experience Scale [133]	1
EFI	Exercise-Induced Feeling Inventory [134,135]	1
FAS	Felt Arousal Scale [136]	1
ES	Ecocentrism scale. Use of natural environments for psychological restoration [137]	1
GEBS	General Ecological Behaviour Scale [138]	1
MUNSH	Happiness Scale based on Memorial University of Newfoundland Scale of happiness [139]	1
Urban HEART-2	Health Equity Assessment and Response Tool-2 (Urban HEART-2) (http://sdh.umin.jp/heart/)	1
HAD	Hospital Anxiety and Depression Scale [140]	1
HPLP-II	Health promoting Lifestyle Profile II [141]	1
ISS	Importance for Survival Scale [142]	1
IWG-2006	International Wellbeing Group 2006. Used to evaluate self-reported, subjective well-being	1
ISEL	Interpersonal Support Evaluation List [143]	1
JSS-N	Job Stress Survey [144]	1
MDI	Major Depression inventory [145,146]	1
MANSA	Manchester Short Assessment of Quality of life [147]	1
MC-SDS	Marlow-Crowne Social Desirability Scale [148]	1
MBI-GS	Korean version of Maslach Burnout Inventory-General Survey [149]	1
MINI	Mini International Neuropsychiatric Interview [150,151]	1
MDBF	Multidimensional Comfort Questionnaire [152]	1
MMS-SF	Multiple Mood Scale-Short form [153]	1
NCQ	Nature Contact Questionnaire [154]	1
NMS	Negative Mood Scale [155]	1
OHI	Oxford Happiness Inventory [156]	1
OHS	Overall Happiness scale [157]	1
PGIS	Personal Growth Initiative Scale [158]	1
PPWB	Physical and Psychological Wellbeing questionnaire [159]	1
PGWB	Psychological General Well-Being Index [160]	1
PRQOL	Influence of parks and recreation on quality of life [161,162]	1
QLCELQ	Quality of Life Concern in End of Life Questionnaire [163,164]	1
QOLI	Quality of Life Inventory (Frisch, 2009). [165]	1
QLS	Quality of Life Scale [166]	1
QLS-ACI	Quality of Life Scale in adults with chronic illness [165]	1
QOLT	Quality of Life tool [167]	1
QEWB	Questionnaire for Eudaimonic Well-Being [168]	1
QEACL	Questionnaires measuring Eudemonia, Apprehension, childhood location. Environments and experiential states. Eliciting participants feelings about place [65]	1
MOS SF-20	Rand medical Outcomes Study Health survey (MOS SF-20) [169,170]	1
RVP	Reason for Visiting the Park, 23-item scale [171,172,173]	1
REP	Recreation Experience Preference scales [174]	1
ROS	Restorative Outcome Scale [175,176]	1
RQE	Restorative quality in environments [65]	1
RCAS	Role conflict and ambiguity scales [177]	1
SMS	Sense of Meaning Scale [178]	1
SCI-93	Stress and crisis inventory [179,180]	1
SEES	Subjective Exercise Experiences Scale [181]	1
BSCS	Self-Control Scale [182]	1
SRRS	Self-rating restoration scale [183]	1
SRSA	Self-reported stress and arousal [184]	1
SOC	Sense of coherence [185]	1
SHAI	Short Health Anxiety Inventory [186]	1
SI-happy	Measuring happiness with a single-item scale [187]	1
SCTS	Social Cohesion and Trust Scale [188]	1
SPS	Social Provisions Scale [189]	1
SWS	Stress at Work Scale by the Behavioural Science Institute, Korea university (1999), occupational stress	1
SRI-MF	Stress response inventory-modified form [190]	1
SRS-18	Stress Response Scale [191]	1
TAP	Taylor Aggression Paradigm [192]	1
TMM	Model of mood [193]	1
CMMS	Current mood measurement scale (The best/worst ever; scale taken from [194]	1
TFI-CS	Therapeutic Factors Inventory–Cohesiveness Scale [195]	1
VQ	Volitional Questionnaire [196]	1
WSRI	Workers Stress Response Inventory; an extended version of the Stress Response Inventory-Modified from [190,197]	1
WOS	Workplace Ostracism Scale [198]	1
WUS	Wildernism-Urbanism Scale [199]	1
ZSDS	Zung self-rating depression scale [200]	1

**Table 8:** Health outcome measure; the most commonly used tools included in studies assessing the associations between GS and mental HWB.

	Paper Number	Primary Health Outcome	Health Outcome Measure
1	[201]	Affect	BDI, SCL-90-R,
2	[202]	Affect	STAI, RRQ, PANAS
3	[203]	Affect	QEACL
4	[204]	Aggression	WOS
5	[205]	Aggression	TAP, BPAQ, BSCS, PRS; PANAS
6	[206]	Anxiety	CSAI-2,
7	[207]	Anxiety	STAI
8	[208]	Chronic stress	PSS
9	[209]	Depression	BDI
10	[210]	Depression	PHQ
11	[211]	Depression	BRFSS, PHQ
12	[60]	Depression	BDI, HAM-17, STAI
13	[212]	Depression	DOQ
14	[39]	Depression	BDI, AFI, BS, PRS
15	[213]	Depression	BDI, STAI, PANAS, PSS, TFI-CS
16	[214]	Depression	IBD, ROS, WEMWBS
17	[215]	Depression	GDS
18	[216]	Depression	GDS
19	[217]	Depression	GDS
20	[218]	Depression	GHQ
21	[219]	Depression	CES-D, DOQ
22	[220]	Depression	MINI, ICD, PSS, WHOQOL
23	[221]	Depression	EPDS
24	[222]	Depression	ZSDS
25	[223]	Depression	CES-D
26	[224]	Depression	BDI
27	[225]	Depression	CES-D
28	[226]	Depression	PHQ
29	[227]	Emotion	POMS
30	[228]	Emotional wellbeing	Urban HEART-2
31	[229]	General health	GHQ
32	[230]	General health	GHQ, POPS
33	[231]	General health	HS SF-36, PSS, DOQ
34	[232]	General health	HS SF-36, PSS
35	[233]	General health	POMS, PANAS, ROS, SVS
36	[24]	General health	POPS, HS SF-36, GHQ,
37	[234]	General health	POPS
38	[235]	General health	HS SF-12,
39	[236]	General wellbeing	RVP, RQE, SWLS, SPNE
40	[237]	General wellbeing	OHI
41	[28]	General wellbeing	EQ-5D, GHQ, DOQ, RSE, POMS
42	[238]	Happiness	SWLS, PANAS
43	[239]	Health anxiety	SHAI
44	[240]	Job stress	SWS
45	[241]	Life satisfaction	DOQ
46	[242]	Life satisfaction	DOQ
47	[243]	Life satisfaction	DOQ
48	[244]	Life Satisfaction	LSIA
49	[245]	Mental health	GHQ
50	[25]	Mental health	GHQ
51	[246]	Mental health	HS SF-36, K10
52	[247]	Mental health	DOQ, CES-D, BAI, RCAS
53	[248]	Mental health	GHQ
54	[249]	Mental health	POPS, GHQ
55	[1]	Mental health	HPLP-II, BDI
56	[250]	Mental health	DASS
57	[251]	Mental health	DASS, MANSA
58	[252]	Mental health	PHQ, PSS, BSI
59	[253]	Mental health	DASS,
60	[254]	Mental health	PANAS, RSE
61	[255]	Mental health	GHQ
62	[256]	Mental health	CES-D
63	[257]	Mental health	PANAS, HAM-17
64	[258]	Mental health	DOQ
65	[259]	Mental health	PS, GHQ, WEMWBS
66	[260]	Mental health	PSQ, GSES, MAAS
67	[261]	Mental health	PS
68	[262]	Mental health	IPA
69	[41]	Mental health	VQ
70	[263]	Mental health	RSE, PSS, POMS
71	[264]	Mental health	MHI-5
72	[265]	Mental health	MHI-5
73	[266]	Mental health	HS SF-12,
74	[267]	Mental health	PS, K10,
75	[268]	Mental health	HS SF-36, DOQ,
76	[269]	Mental health	HS SF-36
77	[270]	Mental health	GHQ
78	[271]	Mental health	DASS
79	[272]	Mental health	GHQ, DOQ
80	[273]	Mental health	WEMEBS, HS SF-12
81	[274]	Mental health	IPA
82	[275]	Mental health	WEMWBS
83	[52]	Mental stress	SRS-18
84	[276]	Mental wellbeing	PS, HS SF-36, K10, BF, DOQ
85	[277]	Mental wellbeing	REP, PAQ
86	[278]	Mental wellbeing	DOQ, WEMWBS,
87	[279]	Mental wellbeing	PSS, WEMWBS
88	[280]	Mental wellbeing	WEMWBS
89	[281]	Mental wellbeing	QOLI, BDI
90	[282]	Mood	RSE, TMD
91	[283]	Mood	TBES, DOQ
92	[284]	Personal development	PGIS, QLS-ACI
93	[285]	Psychological distress	K10
94	[286]	Psychological distress	DOQ
95	[54]	Psychological health	MMSE, GDS, PS
96	[287]	Psychological restoration	ES
97	[288]	Psychological stress	PRS, CN
98	[289]	Psychological wellbeing	ABS
99	[290]	Psychological wellbeing	FS, FAS, MSS
100	[291]	Psychological wellbeing	PRS, PANAS, PSS, CES-D, MUNSH, SPW
101	[292]	Psychological wellbeing	DOQ
102	[293]	Psychological wellbeing	CN, WHOQOL
103	[294]	Psychological wellbeing	STAI, PWB
104	[295]	Psychological wellbeing	SRSMS
105	[55]	Psychological wellbeing	DOQ, GHQ, SCTS, SHCI
106	[296]	Psychological wellbeing	CN, MAAS, FS, SPNE, SVS
107	[297]	Quality of life	PRQOL
108	[298]	Quality of life	EQ-5D
109	[299]	Quality of life	QLCELQ
110	[300]	Quality of life	CRC-QOL
111	[301]	Quality of life	DOQ
112	[302]	Quality of life	LSIA
113	[61]	Quality of life	QOLT
114	[303]	Quality of life	DOQ
115	[62]	Quality of life	QLS
116	[43]	Quality of life	DEMQOL, CS-DD, CMAI, MMSE
117	[304]	Recovery	REQ, DOQ
118	[305]	Restorativeness	EFI, NMS,
119	[306]	Restorativeness	POMS, PRS, NCPC
120	[50]	Restorativeness	ZIPERS, NCPC
121	[50]	Restorativeness	SRRS
122	[307]	Restorativeness	WUS, ZIPERS, OHS
123	[308]	Restorativeness	DOQ, PRS
124	[59]	Restorativeness	DOQ, SRSA
125	[309]	Restorativeness	NCPC, STAI
126	[310]	Restorativeness	PRS, SMBQ, HAD, NCPC, DOQ
127	[142]	Restorativeness	ISS, PRS,
128	[311]	Restorativeness	DOQ
129	[33]	Restorativeness	PRS
130	[312]	Restorativeness	PRS, INS
130	[313]	Restorativeness	PRS, GEBS, MC-SDS,
131	[314]	Restorativeness	WHOQOL, PRS,
132	[315]	Restorativeness	DOQ
133	[316]	Restorativeness	PRS
134	[317]	Restorativeness	DOQ
135	[318]	Restorativeness	PRS
135	[306]	Restorativeness	POMS, PRS, NCPC
136	[319]	Rumination	RRQ
137	[320]	Self-esteem	DOQ
138	[321]	Self-esteem	RSE, POMS, GHQ
139	[63]	Stress	ICD, SMBQ
140	[322]	Stress	PS
141	[323]	Stress	DOQ
142	[53]	Stress	PSS, SPS, HS SF-36
143	[57]	Stress	SRI-MF
144	[58]	Stress	MBI-GS, WSRI, REQ
145	[32]	Stress	CES-D
146	[324]	Stress	PSQ
147	[154]	Stress	PSQ, BRFSS
148	[325]	Stress	DOQ
149	[326]	Stress	MMS-SF, STAI
150	[327]	Stress	DSI, MOS SF-20
151	[328]	Stress	PSS, WEMWBS
152	[329]	Stress	PSS, WEMWBS, PS
153	[37]	Stress	DOQ, SMBQ
154	[330]	Stress	ICD, BM
155	[331]	Stress	SCI-93, EQ-5D,
156	[332]	Stress	PSS, WEMWBS, PS
157	[333]	Stress	PSS, WEMWBS, PS
158	[334]	Stress	ROS, PRS, PANAS
159	[335]	Stress	PANAS
160	[333]	Stress	PSS, WEMWBS, PS
161	[336]	Stress reduction	PANAS, NCPC
162	[337]	Stress reduction	NCQ, QPS, JSS-N, SHCI, DOQ
163	[36]	Stress related mental illness	SMBQ, BDI, BAI, PGWB
164	[338]	Stress restoration	TMM
165	[56]	Stress restoration	PS
166	[246]	Wellbeing	HS SF-36, DOQ
167	[339]	Wellbeing	HS SF-36, K10
168	[340]	Wellbeing	PS
169	[45]	Wellbeing	DOQ
170	[341]	Wellbeing	PRS
171	[342]	Wellbeing	MDBF, SWLS, TPI, HS SF-36
172	[343]	Wellbeing	SVS, UWES, QPS
173	[344]	Wellbeing	SVS, UWES, QPS
174	[345]	Wellbeing	DOQ
175	[346]	Wellbeing	PS
176	[347]	Wellbeing	WEMWBS, CD-RS, SOC, PS, DOQ
177	[348]	Wellbeing	CN, PANAS, SEES
178	[349]	Wellbeing	DOQ, IWG-2006, CN
179	[350]	Wellbeing	MDI, PSS, PANAS, WEMWBS, ISEL
180	[351]	Wellbeing	PWB, PANAS, SWLS, ES-SF
181	[352]	Wellbeing	PANAS, EES, SMS, CN
182	[353]	Wellbeing	DOQ, OHI, INS
183	[354]	Wellbeing	CN, PRS, PAQ, PANAS, PPWB
184	[355]	Wellbeing	STAI, PRS
185	[356]	Wellbeing	CN, QEWB, WHOQOL
186	[357]	Wellbeing	DOQ
187	[358]	Wellbeing	PANAS, INS

**Table 9:** Overview of the ten most used health endpoints and the tools most commonly used to assess them – the number next to the tool is the number of studies where it was used.

Mental Health		Wellbeing		Stress		Restorativeness		Depression		Quality of Life		Psychological Wellbeing		General Health		Mental Wellbeing		Life Satisfaction	
GHQ	8	DOQ	7	PSS	6	PRS	11	BDI	5	DOQ	2	DOQ	2	GHQ	3	WEMWBS	3	DOQ	3
DASS	4	PANAS	6	PS	5	DOQ	6	CES-D	3	CS-DD	1	CN	2	POPS	3	DOQ	2	LSIA	1
DOQ	4	CN	5	WEMWBS	5	NCPC	5	GDS	3	CMAI	1	FS	2	HS SF-36	3	BDI	1		
HS SF-36	3	HS SF-36	3	DOQ	3	POMS	2	PHQ	3	CRC-QOL	1	ABS	1	PSS	2	BF	1		
PS	3	PRS	3	ICD	2	ZIPERS	2	DOQ	2	DEMQOL	1	CES-D	1	DOQ	1	HS SF-36	1		
RSE	2	PS	3	PANAS	2	EFI	1	PSS	2	EQ-5D	1	FAS	1	HS SF-12	1	K10	1		
WEMWBS	2	INS	2	PSQ	2	HAD	1	PHQ	2	LSIA	1	GHQ	1	PANAS	1	PAQ	1		
CES-D	2	QPS	2	SMBQ	2	ISS	1	STAI	2	MMSE	1	MSS	1	POMS	1	PS	1		
HS SF-12	2	SVS	2	BM	1	NMS	1	AFI	1	QLCELQ	1	MUNSH	1	ROS	1	PSS	1		
IPA	2	SWLS	2	BRFSS	1	SMBQ	1	BRFSS	1	QLS	1	PANAS	1	SVS	1	QOLI	1		
K10	2	UWES	2	CES-D	1	SRRS	1	BS	1	QOLT	1	PRS	1			REP	1		
MHI-5	2	WEMWBS	2	DSI	1	SRSA	1	EPDS	1			PSS	1						
PANAS	2	CD-RS	1	EQ-5D	1	STAI	1	GHQ	1			PWB	1						
PSS	2	EES	1	HS SF-36	1			IBD	1			SCTS	1						
BAI	1	ES-SF	1	MBI-GS	1			ICD	1			SHCI	1						
POMS	1	ISEL	1	MMS-SF	1			MINI	1			SPW	1						
BSI	1	IWG-2006	1	MOS SF-20	1			PANAS	1			SRSMS	1						
GSES	1	K10	1	PRS	1			PRS	1			STAI	1						
HPLP-II	1	MDBF	1	REQ	1			ROS	1			SVS	1						
MANSA	1	MDI	1	ROS	1			WEMWBS	1			WHOQOL	1						
MAAS	1	OHI	1	SCI-93	1			WHOQOL	1										
PHQ	1	PAQ	1	SPS	1			ZSDS	1										
POPS	1	PPWB	1	SRI-MF	1														
PSQ	1	PSS	1	STAI	1														
RCAS	1	PWB	1	WSRI	1														
VQ	1	QEWB	1																
		SEES	1																
		SMS	1																
		SOC	1																
		STAI	1																
		TPI	1																
		WHOQOL	1																

**Table 10:** Randomized controlled trials investigating the effects of GS on mental health.

**Paper Number**	**Country/Green Space**	**Participant Type**	**# of Subjects/Male/Female**	**Mean Age/min/max**	**Positive/Negative**	**Health Outcome**	**Health Assessment**	**Green Space Assessment**	**Quantitative/Qualitative Study Methods**	**Intervention/Control Group**	**Comments**
**2-arm randomised controlled crossover design**											
[50] Study 2 *	UK/Natural	University students	17/7/10	23.18 (±6.23)/18/43	Mixed/Negative	Restorativeness	Questionnaire, heart rate	Photos, videos. No in-depth quality assessment, description, or quantitative measures	Quantitative	Walk through low prospect-high refuge natural environment/Walk through high prospect-low refuge environment	Small study with indication the GS is restorative only when there is an open aspect and few places where someone might hide.
[361]	USA/Urban	General public	12/8/4	x/x/x	Positive	Stress, Health	Heart rate	Observations. No quantitative assessment	Quantitative	Self-paced walk in local neighbourhood past sites receiving greening treatment/Self-paced walk in local neighbourhood past sites not receiving greening treatment	Small study only measuring heart rate. Based on heart rate only, the results indicated that in-view proximity to a greened vacant lot decreased heart rate compared to in-view proximity to a non-greened vacant lot.
[57]	Korea/Natural	University students	41/14/27	x/18/35	Positive	Stress	Cytokine serum levels, questionnaire	No quantitative or qualitative assessment	Quantitative/Qualitative	2 h exposure to a forest environment/2 h exposure to an urban environment	Small study indicating the level of somatic and depressive symptoms decrease significantly after exposure to forest environments. Weak design; no before-and-after measurements allowing for comparison.
[57]	Japan/Natural	Male university students	12/12/0	21.3 (±1.1)/20/23	Mixed/Negative	Restorativeness	Cortisol, blood pressure, pulse rate, questionnaire	No quantitative or qualitative assessment	Quantitative/Qualitative	15 min visits to forest environments/15 min visits to urban environments	Small study with no clear conclusion from the quantitative data about the effect of GS. Subjective evaluation data showed significantly more positive responses after exposure to forest environments.
[326]	Japan/Natural	General public	498/244/254	56.2 (±10.6)/20/x	Positive	Stress, Mental health	Questionnaire	No quantitative or qualitative assessment	Qualitative	2 x forest walks/2 days where a forest was not visited	Relatively large number of participants, but no quantitative objective data. The study concluded that a forest environment significantly reduces hostility and depression. The largest benefit was seen for the most stressed participants.
[318]	Sweden/Virtual, indoor	Undergraduates	74/x/x	x/x/x	Positive	Restorativeness	Questionnaire	852 colour photos of two gardens were sampled. Final sample consisted of 12 photos for each garden.	Qualitative	2 ha large spacious garden with large as well as small garden rooms and many views without buildings/Small and detailed courtyard garden of 13 × 17 m. Views at eye-level always include buildings.	A study using only one qualitative measure and no quantitative data. The data showed that gardens are likely to be restorative to varying degrees, depending on the design and the surroundings of the garden.
[233]	Japan/Urban	Undergraduates	45/45/0	21.13 (±1.25)/x/x	Positive	General health	Questionnaire	Photos. No in-depth quality assessment, description, or quantitative measures	Qualitative	A 15 min walk in a forest environment/A 15 min walk in an urban environment	Relatively small study looking only at young men. Four different validated questionnaires were used; some revealed significant positive effects of the forest environment, some did not. The results indicated the combination of activity and GS results in greater psychological benefits. The feelings of vigour, positive effects, subjective recovery, and vitality were stronger in the forest environment.
[209]	USA/Urban	People with depression	20/8/12	26/x/x	Mixed/Negative	Cognition, Affect	Interview, questionnaire	Satellite GPS images. No in-depth quality assessment, description, or quantitative measures	Qualitative	50 min walk in natural setting/50 min walk in urban setting	Small study with no clear conclusions about the effect of GS.
**2-arm randomised controlled design, no crossover**											
**Author**	**Country/Green space**	**Participant type**	**# of subjects/male/female**	**Mean age/min/max**	**Positive/Negative**	**Health outcome**	**Health assessment**	**Green space assessment**	**Quantitative/Qualitative**	**Intervention/Control group**	**Comments**
[60]	Korea/Natural	Patients with history of stroke	59/40/19	60.8 (±9.1)/36/79	Positive	Depression. anxiety	Questionnaire, physiological measurement (Reactive oxygen metabolites (dROM). Biological antioxidant potential (BAPs))	No in-depth quality assessment, description, or quantitative measures	Quantitative/Qualitative	Patients randomly assigned to a forest therapy group or an urban control group.	The study found that forest therapy can significantly lower oxidative stress and improve anti-oxidative capacity for patients with a history of stroke. High levels of oxidative stress and reduced anti-oxidative capacity are indicative of depression and anxiety.
[306]	China/Urban	College students	32/16/16	20.6 (±1.6)/x/x	Positive	Cognition, restorativeness	Questionnaire, EEG	Photos. Quantification of green elements, buildings and paved areas of the two environments used.	Quantitative/Qualitative	20 min exposure to one of two environments: A wooded campus garden/A traffic island under an elevated highway	Positive EEG results identified from a brief exposure to photographs of nature compared to urban environment (20 min).
[319]	USA/Urban	General public	30/14/16	26.6/x/x	Positive	Rumination	Questionnaire, neural activity in the sgPFC	Detailed description of the two walk.	Quantitative/Qualitative	5.3 km nature walk/5.3 km urban walk	The study found a significant reduction in self-reported rumination driven by a decreased cerebral blood flow in the sgPFC for the nature group, but not for the urban group.
[335]	Netherlands/Horticulture, garden	General public	30/8/22	57.6 (±8.49)/38/79	Positive/mixed	Stress	Questionnaire, salivary cortisol	Very brief description of allotment complex but no in depth description or quantitative measures of the GS investigated	Quantitative/Qualitative	After stress induction: 30 min of outdoor gardening in own allotment/30 min of indoor reading in allotment home with no view of nature (popular magazines chosen by researcher)	The small study found that both reading and gardening showed a significant reduction in cortisol levels after stress. Cortisol levels were lower after gardening compared to reading, but the difference was not significant. Positive mood was significantly higher after gardening compared to reading. There were indications that gardening is more restorative after stress than reading.
[204] – study 1	USA/Virtual, indoor	General public	86/22/64	35.47 (±14.05)/x/x	Positive	Aggression	Questionnaire		Qualitative	Ostracised individuals exposed to urban or nature pictures/Non-ostracised individuals exposed to urban or nature pictures	The qualitative study found that among participants with a high feeling of ostracism, those who viewed nature pictures reported a significantly lower level of aggression than those who viewed urban pictures. The authors concluded that nature exposure can counteract the relationship between ostracism and aggression.
[289]	USA/Virtual, indoor	Older adults	62/6/56	x/x/x	Mixed, negative	Physiological wellbeing	Questionnaire	No in-depth quality assessment, description, or quantitative measures	Qualitative	Horticultural activity program in a care home, once a week for 7 weeks/Normal daily activities in a care home, over 7 weeks	The study found no statistically significant differences in the effect of a horticultural activity program on physiological wellbeing of older adults in a care home. However, there were some indications that horticultural activities may have a positive effect on wellbeing.
[202]	USA/Urban	General public	60/27/33	22.9/x/x	Mixed	Affect, cognition	Questionnaire	Photos. No in-depth quality assessment, description, or quantitative measures	Qualitative	A nature walk/An urban walk	The study found significant evidence that a nature walk improves affect, but no clear evidence that it improves cognition.
[324]	USA/Urban	University office staff	37/34/3	48.8/x/x	Positive	Stress	Questionnaire	No description or quantitative measures of the GS investigated.	Qualitative	Work breaks over 4 weeks: 10–15 min outdoor booster break/standard work break	The small qualitative study found that a 10–15 min outdoor booster break during the work day results in a significantly greater reduction in stress than an indoor work break.
[320]	USA/Horticulture, garden	Undergraduates	32/x/x	x/18/32	Mixed/Negative	Self-esteem	Questionnaire	No in-depth quality assessment, description, or quantitative measures	Qualitative	4 h of gardening work over a period of 3 weeks/No gardening activities	This small qualitative study found no significant differences regarding ethnocentrism and self-esteem, in relation to the effects of GS. There were indications that gardening can positively affect self-esteem.
[271]	Serbia/Horticulture, garden	Psychiatric patients	30/9/21	45.35 (±10.16)/25/65	Mixed	Mental health	Questionnaire	Map, photos and short description. No in-depth quality assessment or quantitative measures	Qualitative	Four weeks (12 sessions) of horticultural therapy/Four weeks of occupational art therapy	The small qualitative study found a significantly larger reduction in stress after horticultural therapy compared to occupational art therapy. However, no significant differences were identified for anxiety or depression after the two treatments.
**3-arm randomised controlled design, no crossover**											
**Author**	**Country/Green space**	**Participant type**	**# of subjects/male/female**	**Mean age/min/max**	**Positive/Negative**	**Health outcome**	**Health assessment**	**Green space assessment**	**Quantitative/Qualitative**	**Intervention/Control group**	**Comments**
[307]-Study 2 **	USA/Urban	College students	34/17/17	20/x/x	Positive	Restorativeness	Questionnaire, physiological measurements (blood pressure and pulse)	Very brief description of GS	Quantitative/Qualitative	College students were randomly assigned to a nature walk, an urban walk, or a relaxation condition	Small study showing that happiness and positive affect significantly increase and anger and aggression significantly decreased after being in a natural environment compared to an urban environment.
[304]	Finland/Urban	Office workers	153/137/20	47.2/x/x	Mixed	Stress, wellbeing	Questionnaire (paper format and mobile text messages)	No in-depth quality assessment, description, or quantitative measures	Qualitative	Park walk/relaxation exercises/usual break activities	The study found no clear conclusions about the effect of park walks on employees’ wellbeing. The effects on wellbeing were of a small magnitude and short duration.
[338]	USA/Urban	Park visitors	108/48/60	22/x/x	Mixed, negative	Stress reduction, restorativeness	Questionnaire	No in-depth quality assessment, description, or quantitative measures	Qualitative		
[282]	UK/Urban	Adults with mental health issues	53/20/33	53 (±15.4)/21/83	Mixed	Mood, self-esteem	Questionnaire	No in-depth quality assessment, description, or quantitative measures	Qualitative	Three health-promoting interventions: Walking in GS/Swimming/Quizzes, bingo, games, crafts and music	The study found that green exercise was as health-promoting for people experiencing mental ill health as existing non-green interventions. There was no conclusive evidence that GS activity was more health-promoting than other activities.
[217]	USA/Horticulture, garden	Older adults with mild to moderate depression	39/16/23	74.3 (±6.40)/x/x	Positive	Depression	Questionnaire, focus groups	No in-depth quality assessment, description, or quantitative measures	Qualitative	The participants were randomly assigned to one of 3 treatments; walk alone, guided imagery, or art therapy.	Small study surmising that GS as well as art interventions were helpful in improving mood and overall attitude. However, only subjective, anecdotal evidence was explored.
[215]	USA/Horticulture, garden	Older adults with mild to moderate depression	39/x/x	75/x/x	Positive/Mixed	Depression	Questionnaire, focus groups	No in-depth quality assessment, description, or quantitative measures	Qualitative	The participants were randomly assigned to one of 3 treatments; walk alone, group walking, or art therapy.	The study found that assisted and unassisted GS walks as well as art therapy interventions can significantly reduce symptoms of depression.
[336]	Iceland/Urban	University students	18/9/9 ***	x/x/x	Mixed/negative	Stress	Interviews, observations	Photos. No in-depth quality assessment, description, or quantitative measures	Qualitative	Three treatments for alleviation of stress: Walking in the gym/Walking in nature/Watching nature on TV	The very small study using personal narratives involving restoration found no clear conclusions about the effect of GS on stress.
**4-arm randomised controlled crossover design**											
**Author**	**Country/Green space**	**Participant type**	**# of subjects/male/female**	**Mean age/min/max**	**Positive/Negative**	**Health outcome**	**Health assessment**	**Green space assessment**	**Quantitative/Qualitative**	**Intervention/Control group**	**Comments**
[310]	Sweden/Natural	Females diagnosed with exhaustion disorder	20/0/20	41.6 (±7.3)/24/55	Positive	Restorativeness	Questionnaire, heart rate, blood pressure, heart rate recovery	Photos, detailed description. No quantitative assessment.	Quantitative/Qualitative	90 min test procedure in 3 different forest environments/and in 1 city environment	Small study indicating significantly higher perceived restorativeness in the forest environments compared to the city.
**4-arm randomised controlled design, no crossover**											
**Author**	**Country/Green space**	**Participant type**	**# of subjects/male/female**	**Mean age/min/max**	**Positive/Negative**	**Health outcome**	**Health assessment**	**Green space assessment**	**Quantitative/qualitative**	**Intervention/Control group**	**Comments**
[227]	Taiwan/Urban	College students	116/52/64	20.85 (±1.14)/x/x	Mixed/Negative	Emotion, attention	Questionnaire	Photos used to quantify the level of greenness, aerial maps	Quantitative/Qualitative	Walking or jogging in natural environment/Walking or jogging in built environment	The study found no clear conclusions about the effect of GS and exercise on emotion and attention. The key finding is the indication that walking in a setting with at least 40% visible greenness elicits the largest benefits.
[204] – study 2	USA/Virtual, indoor	General public	150/48/102	36.87 (±13.30)/x/x	Mixed	Aggression	Questionnaire	Photos. No in-depth quality assessment, description, or quantitative measures	Qualitative	Ostracised individuals exposed to urban or nature pictures/Non-ostracised individuals exposed to urban or nature pictures	The study found no clear conclusions about the effect of viewing nature photos to moderate the relationship between ostracism and aggression. There were some indications that viewing nature photos can alleviate aggressive responses following ostracism.
[204] – study 3	USA/Virtual, indoor	General public	144/47/97	35.47 (±11.99)/x/x	Mixed	Aggression	Questionnaire	Photos. No in-depth quality assessment, description, or quantitative measures	Qualitative	Ostracised individuals exposed to urban or nature pictures/Non-ostracised individuals exposed to urban or nature pictures	The study found no clear conclusions about the effect of viewing nature photos to moderate the relationship between ostracism and aggression. There were some indications that viewing nature photos can alleviate aggressive responses following ostracism.
[205]	China/Virtual, indoor	Undergraduate students	118/25/93	21.23 (±2.26)	Mixed, negative	Aggression, mood	Questionnaire	Video. No in-depth quality assessment, description, or quantitative measures	Qualitative	Depleted individuals exposed to a natural or urban video/Non-depleted individuals exposed to a natural or urban video	The study found no clear conclusions about the effect of viewing a natural video to counteract aggression after depletion. The study suggests that watching a natural video helps to restore self-control after depletion.

x = data missing. ± = standard deviation around the mean. * This paper consists of three small studies; only one of which is presented in this table (study 2). ** This paper consists of two studies; only study 2 is presented in this table. *** Only three participants are described in the results; one for each treatment.
